# Sensor to Monitor Localized Stresses on Steel Surfaces Using the Magnetostrictive Delay Line Technique

**DOI:** 10.3390/s19214797

**Published:** 2019-11-04

**Authors:** Kaiming Liang, Spyridon Angelopoulos, Georgios Lepipas, Panagiotis Tsarabaris, Aphrodite Ktena, Xiaofang Bi, Evangelos Hristoforou

**Affiliations:** 1 Key Laboratory of Aerospace Materials and Performance (Ministry of Education), School of Materials Science and Engineering, Beihang University (BUAA), Beijing 100191, China; liangkm@buaa.edu.cn; 2 School of Electrical & Computer Engineering, National Technical University of Athens, Zografou Campus, 15780 Athens, Greece; spyrosag@central.ntua.gr (S.A.); giwrgoslep@gmail.com (G.L.); ptsarab@central.ntua.gr (P.T.); 3 Energy Systems Laboratory, National & Kapodistrian University of Athens, Evripos Campus, 34400 Evia, Greece; ktenaa@gmail.com

**Keywords:** force sensor, localized residual stress, surface permeability

## Abstract

In this paper, a new type of force sensor is presented, able to monitor localized residual stresses on steel surfaces. The principle of operation of the proposed sensor is based on the monitoring of the force exerted between a permanent magnet and the under-test steel which is dependent on the surface permeability of the steel providing a non-hysteretic response. The sensor’s response, calibration, and performance are described followed by a discussion concerning the applications for steel health monitoring.

## 1. Introduction

Monitoring stresses on the surface and in the bulk of metals, and particularly steels, is essential in the health monitoring of metal structures. Residual stresses are usually introduced during manufacturing, may be desired or undesired, and may enhance or decrease the quality of the steel depending on the required properties and intended use [1,2]. Information on residual stresses and particularly the residual stress gradient may be used to predict crack initiation and propagation [3]. Typical industrial methods to monitor stresses are the strain gauge principle [4] and the hole-drilling method [5]. They both monitor micro-strain changes and therefore provide a measure for the residual stress gradient. The strain gauge can monitor local micro-strains once it is attached to the under-test surface, but the preparation of the surface to host the strain gauge is laborious and tedious. The hole-drilling method provides vector stress monitoring, but it is a more or less destructive monitoring method. Several sensors, like piezoelectric and electromagnetic-acoustic transducer sensors (EMAT), have also been implemented for crack detection and magnetic properties characterization [6,7].

Apart from these sensing methods, the response of other suitable sensors and instruments can be correlated and calibrated against stress measurements obtained by laboratory instruments. Surface stresses can be determined using X-Ray diffraction according to the Bragg-Brentano (XRD-BB) method [8]. The XRD-BB method can monitor average stresses on a surface from 1 to 4 mm^2^, depending on the X-ray beam diameter, with an uncertainty minimized down to 1% [9]. Bulk stresses can be monitored by using neutron diffraction [10], following a similar procedure as in the XRD-BB method. The best spatial resolution of the neutron diffraction method is in the order of 5–10 mm^2^, while uncertainty is usually in the order of 2–5%.

Sensing elements able to monitor surface stresses in steels can be those monitoring localized changes of sound velocity, including the use of non-linear acoustics [11], electromagnetic properties such as eddy currents for resistivity and impedance determination [12], optical properties using the Kerr effect [13], as well as magnetic properties like surface permeability in the magnetic stress calibration curve (MASC) technique [14] and the magnetic memory method (MMM) [15], or magnetic Barkhausen noise method [16]. The sensing elements used in acoustic techniques offer a spatial resolution in the order of cm and may be used in industrial conditions. Eddy current sensors offer a resolution of mm, however, their response is correlated to cracks and not residual stress distribution, due to the dynamic, as opposed to static, excitation field used. Optical sensors can offer a resolution of μm, but they are usable only in laboratory conditions; magnetic permeability sensors offer a spatial resolution in the order of mm and can monitor stresses in an industrial environment. Barkhausen noise sensors offer a spatial resolution of mm, but they suffer from low repeatability and high uncertainty.

Techniques monitoring bulk stresses mainly utilize acoustic sensors and magnetic permeability sensors [17,18]. Therefore, targeting the mm spatial resolution and industrial use of the sensors for surface and bulk stress monitoring, our group has opted to monitor the differential permeability and to correlate these measurements with either the surface [19] or bulk [20] stress gradient. Previous works have demonstrated that permeability measurements can reach mm spatial resolution and 1% uncertainty [9].

**Figure 1:**
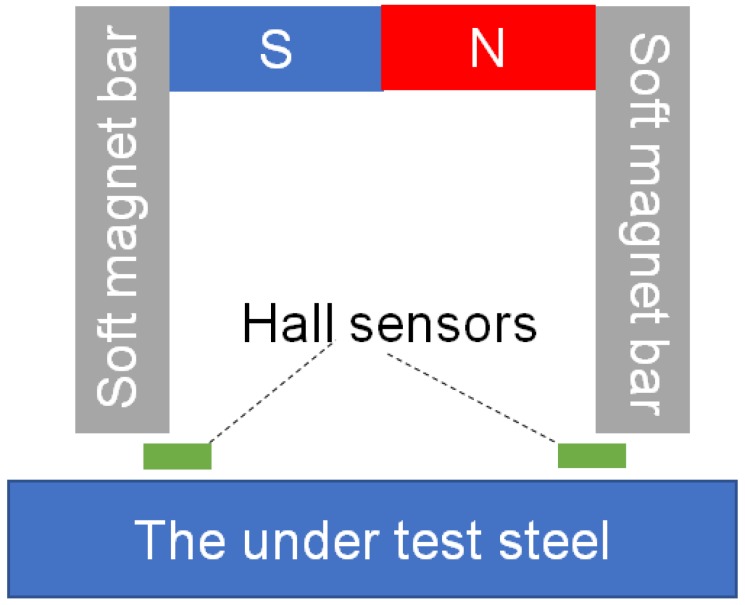
The permanent magnet yoke arrangement for ambient field compensation.

The rather classic measurement technique for the monitoring of magnetic permeability is based on yoke arrangements of various designs [21]. The typical yoke arrangement suffers from the usual problem of all magnetic sensors, namely the effect and interference of the ambient magnetic field on the output of the yoke, and therefore on the resulting localized permeability measurement. A yoke arrangement that decreases or even eliminates the effect of the ambient field is depicted in Figure 1: The horizontal part of the yoke is made of a permanent magnet bar while the legs are made of two soft magnetic bars—a pair of Hall sensors connected in series opposition, firmly set at a given distance underneath the two soft magnetic bars, are used to monitor the magnetic flux entering and leaving each leg. When a permanent magnet yoke is firmly fixed at a distance from the under-test steel (Figure 1), the Hall sensors monitor the path of the magnetic flux lines from the yoke to the under-test steel, which monotonically depends on the permeability of the under-test steel at the vicinity of the yoke legs. The outputs of the two Hall sensors monitor the same ambient field, and the in series-opposition connection cancels it from their collective output. The resolution of such a sensor is in the order of cm, while mm resolution can be obtained only by mm-range yoke displacement. Apart from that, if rapid monitoring is needed, the magnetic flux entering the under-test steel is not static anymore, which leads to eddy current effects and misleading readings at the Hall sensor output.

The motivation of this paper has been the development of a new alternative solution to monitor stresses in the under-test steel, overcoming the limitations of the permanent magnet yoke described above. The development of a force sensor to monitor the magnetic force generated between a permanent magnet and the under-test steel is proposed when the permanent magnet is firmly set on top of it, taking advantage of the proportional dependence of this force on the permeability of the under-test steel. The foreseen advantage of this is the controllable spatial resolution, dependent on the size of the permanent magnet and the force sensor. Another advantage is the negligible effect of small ambient fields because of the much higher magnetic flux density in the under-test steel generated by the permanent magnet. In this paper, the basic assumptions and design considerations for such a sensor are outlined and experimental results and discussion on the sensor response and applications are presented.

## 2. The Sensor

The correlation between residual stresses and differential permeability (in the remaining of the paper, referred to as permeability for simplicity reasons), has been well established in the past. It has been concluded that the dependence of surface or bulk permeability on the surface or bulk residual stresses, respectively, is a monotonic function, permitting uncertainty levels in the order of 1% [9].

The main idea presented in this work is the development of a sensor to monitor the force between a permanent magnet and the under-test steel and the correlation of this force directly with the localized permeability of the steel in the region affected by the permanent magnet. Such force is in the order of 5–10 N [21]. Therefore, force sensors operating within this span may be suitable for monitoring this force.

**Figure 2:**
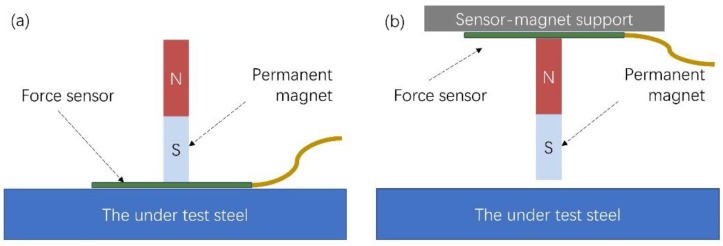
The schematic of the force sensor: (**a**) The force sensor is between the permanent magnet and the under-test steel; (**b**) the permanent magnet is between the force sensor and the under-test steel.

This force can be measured by fixing the permanent magnet-force sensor assembly on robust support, allowing for the precise control of the distance between the permanent magnet and the under-test steel in order to avoid lift-off effects. Thus, the force sensor attached to the magnet can detect the magnetic force between the permanent magnet and the under-test steel. Provided that the force sensor output is monotonically related to the localized permeability of the under-test steel. Then, the output is also correlated to the localized residual stresses of the steel. The schematic of the sensing element is illustrated in Figure 2a. A similar arrangement can be implemented by suspending the force sensor–permanent magnet arrangement from robust support, maintaining the distance between the permanent and the under-test steel well controlled (see Figure 2b). The advantage of the sensor of Figure 2a is the firmly fixed lift-off of the permanent magnet-force sensor assembly from the under-test steel, with the disadvantage of the contact between the sensor and the under-test steel. The advantage of the sensor of Figure 2b is the contactless monitoring of force, with the lift-off being a possible issue. In both sensors shown in Figure 2, a displacement sensor could be used to monitor the position of the permanent magnet–force sensor assembly and allow for gradient force monitoring.

**Figure 3:**
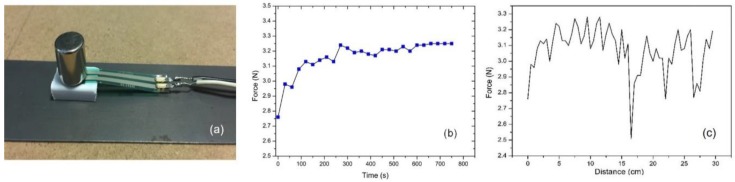
The FSR sensor used as a permeability sensor: (**a**) the sensor arrangement, where the FSR is between the permanent magnet and the under-test steel; (**b**) the time response of the FSR sensor; (**c**) indicative surface scanning of a non-oriented electric steel.

Initially, the commercially available FSR sensing element (FSR 400, Interlink Electronic) [22] was used, as illustrated in Figure 3a, due to the simplicity of the whole arrangement. This force sensor is based on a capacitive element, whose capacitance increases or decreases with the applied force. Due to the relatively small force range of the FSR sensor, the distance between the permanent magnet and the under-test steel was intentionally increased using a ceramic rectangular piece, to decrease the force exerted between the permanent magnet and the steel. However, the time response of the FSR, illustrated in Figure 3b, is in the order of several hundreds of seconds, thus making the use of the sensor impractical for fast measurements. Apart from that, the stability of the sensor was not at the desired levels. As an example, when the sensor was used to scan the surface of non-oriented electric steel which, based on the material’s magnetic and structural characterization, was expected to give a rather homogeneous response, the force sensor response (Figure 3c) was far from the expected results.

Other types of similar force sensors, like piezoelectric sensors [6], haven’t been implemented for similar reasons: the time response doesn’t permit a fast monitoring process.

To address the above shortcomings and provide a proof of principle for the proposed magnetic force-based non-destructive testing method, the commercially available FSR sensor could be replaced by other types of magnetic force detection, like EMAT sensors [7] or magnetostricitive delay lines (MDL) force sensors [23]. The MDL technique was chosen for the implementation of the new force sensor, due to the specialization of the lab in this technique. The MDL principle utilizes an excitation coil fixed at a given volume around a magnetostrictive ribbon with a uniform cross-section, the MDL, which acts as an acoustic waveguide. Pulsed current is transmitted through the excitation coil, and therefore a pulsed magnetic field is generated along the length of the MDL at a volume approximately equal to the volume of the MDL enclosed by the excitation coil. A pulsed microstrain is consequently generated due to the magnetostriction effect within this volume. The microstrain propagates in both directions of the MDL as a Lamb wave. As the microstrain pulse propagates within the volume of the MDL, it causes pulsed magnetic flux changes within these volumes, due to the inverse magnetostriction effect. A search coil set at a distance from the excitation coil, long enough to avoid interference between excitation and detection signal, can detect the propagating pulsed microstrain in the form of pulsed voltage output, proportional to the first derivative of the magnetic flux change within the volume of the search coil. Practically, the distance between the excitation and search coil should be longer than the time duration t of the pulsed voltage output multiplied by the longitudinal sound velocity υ of the MDL. Bearing in mind that t and υ are 14 μs and 5 km/s respectively, the distance between the excitation and search coil should be longer than 70 mm. The peak amplitude of the pulsed voltage output, Vo, is used as the MDL output. 

Two types of MDL force sensors have been developed in the past, namely a force sensor [23] based on the distortion of the propagating microstrain and the resulting decrease of Vo by the force applied on the MDL between the excitation and the search coils, and a force digitizer [24] based on the acoustic reflection generated inside the MDL when the force is applied on a point of the MDL outside the volumes enclosed by the excitation and search coils. It is noted that the response of both sensors is exponential with respect to the applied force. The force sensors developed in this work to monitor the residual stress dependent force and, consequently, permeability, are based on these two MDL designs and are analyzed next.

**Figure 4:**
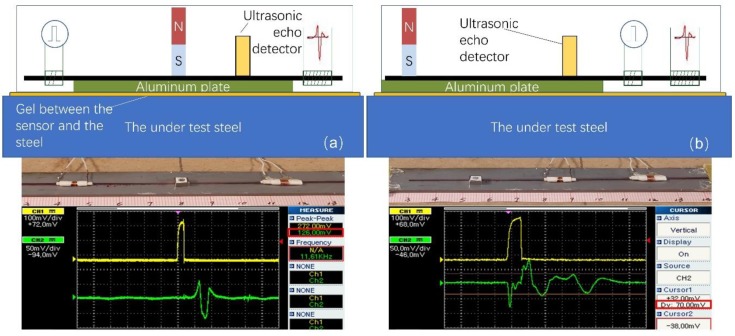
The force-permeability sensor based on the magnetostrictive delay line (MDL) principle: (**a**) the force sensor monitors the permeability changes as a result of the distortion of elastic propagating waves, observed by the decrease of the Vo with the exerted force; (**b**) the sensor monitors the permeability changes as a result of the reflections generated in the volume where the permanent magnet is pressing the MDL, observed by the Vo of the second detected pulse, corresponding to the reflection mentioned above.

The surface permeability sensor based on force measurements and the force digitizer principle is illustrated in Figure 4. According to this arrangement, an aluminum plate is set as the basis of the sensor, which may be in contact with the under-test steel, to provide the necessary distance between this and the permanent magnet, according to the force span of the MDL. Experimenting with different thicknesses of the non-magnetic plate, it was concluded that 3–5 mm thickness of aluminum used for a 5 × 5 mm cross-section area of the permanent magnet and ~1 mm thick low carbon steel resulted in forces between the magnet and the steel within the range of both sensors, i.e., ~5N−10N.

A Fe_78_Si_7_B_15_ amorphous magnetostrictive ribbon is single-sided glued by acrylic glue on the aluminum plate, serving as the MDL, as single-side MDL glue permits the propagation of the elastic microstrain. For practical, indicative, and not exclusive reasons, the width, length, and thickness of the ribbon are 6 mm, 100 mm, and 25 μm, respectively. On top of the ribbon a 10 mm long Nd-Fe permanent magnet of rectangular cross-section 5 × 5 mm^2^ is glued by acrylic glue, in the middle of the amorphous ribbon, thus forming the force sensor (Figure 4a), or at one end of it, forming the force digitizer (Figure 4b). In both sensing elements, the 0.5 mm long excitation coil, made of 10 turns of 0.2 mm enameled Cu wire, is set at the one free end of the ribbon. In the case of the force sensor (Figure 4a), the 300 turns and 2 mm-long search coil made of 0.05mm enameled Cu wire is set at the other end of the ribbon, while in the case of the force digitizer (Figure 4b), it is set 30 mm from the permanent magnet or 70 mm from the excitation coil. In both sensors, the sensing element is the assembly of permanent magnet-ribbon-aluminum.

**Figure 5:**
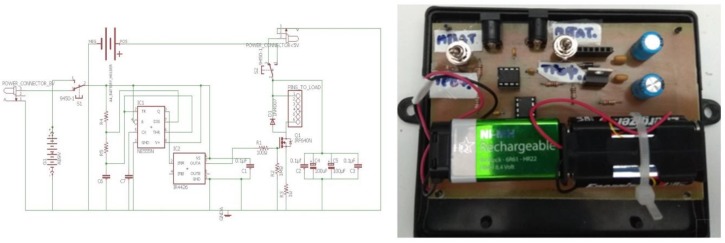
The electronic circuit for the excitation and the detection of the propagating pulsed microstrain of the MDL force sensor.

The excitation circuit is a high bandwidth power MOSFET able to provide a pulsed current of 0.1 A to 30 A, with 1–2 μs duration and 1 ms period. The actual current amplitude is fixed to 8 A, enough to generate a pulsed voltage output V_oa_, in the order of 100 mV at the output of the search coil. The output signal of the search coil is driven to an operational amplifier of gain from 400–1000 and then to a peak holder buffer, driven to a 12 bit A to D converter, with time clock at 30 kHz. The schematic of the electronics is illustrated in Figure 5. The sensor packaging at this stage includes the resin cover of the assembly described above, including electronics. At this stage, the digitized information is wired to the data storage bank, while in the future the system will be equipped with a wireless communication system.

Before the sensor operation, a pulsed current of a pre-selected amplitude is transmitted to the excitation coil, and the maximum output voltage, Vo, for both sensors is monitored as a reference value. As the sensing element is attached to the under-test steel surface, the electronic circuit starts recording the voltage output, corresponding to the force exerted between the permanent magnet and the under-test steel. For the case of the force sensor (Figure 4a), the peak to peak voltage output Vo decreases with the applied force, or with the localized permeability of the under-test steel, because the elastic reflections generated at the area of the exerted force result in a decrease of the elastic wave picked up by the search coil, which is proportional to the applied force. For the case of the force digitizer (Figure 4b), the peak voltage output Vo at the reflection point corresponding to the position of the permanent magnet increases with the applied force on the under-test steel, or with the localized permeability of the under-test steel.

## 3. Experimental

Previous work has already shown that stress monitoring may be achieved through the monitoring of the localized permeability of the under-test steel [9,14,19,20]. In this work, we show that instead of a permeability sensor, a force sensor may be used to avoid the drawbacks of a yoke-type permeability sensor which would be an appropriate solution for industrial use. The response of the sensing elements of Figure 4a,b is due to the force exerted by the permanent magnet on the MDL. The force, and therefore the output voltage peak, depends on the localized permeability of the under-test steel. Therefore, it is preferable to calibrate the sensor directly with the permeability of the under-test steel. In this section we present the method to determine the dependence of the sensor on the permeability of the under-test steel, having sorted out manually the lift-off effect.

Three different types of steel have been tested, namely 0.35 mm thickness oriented electric steel (OES), 0.25 mm thickness non-oriented steel (NOES), and 0.5 mm low carbon steel (LCS). The design of the sensor permits the same dependence on permeability for different types of steels, provided that their localized surface permeability and thickness are assumed to be identical.

The calibration of the sensor with respect to the permeability of the under-test steel was performed in the following way: (i) determination of permeability dependence on field of the under-test steel(s) using a primary standard, (ii) calibration of a transfer standard, and finally (iii) calibration of the sensor dependence on permeability using the transfer standard.

**Figure 6:**
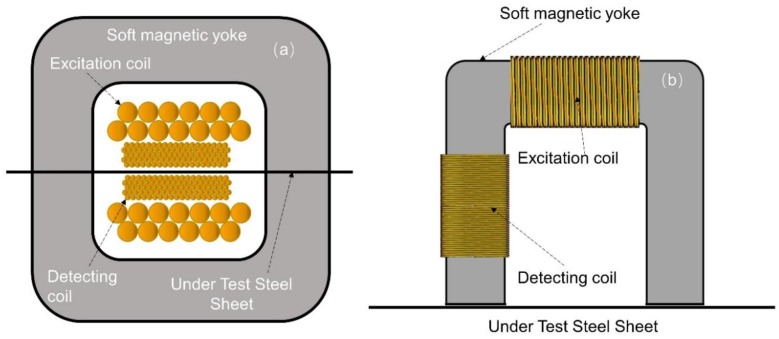
The permeability measurement set-ups: (**a**) the primary permeability tester based on the single sheet standard method; (**b**) the secondary standard based on a yoke arrangement magnetizing the surface of the under-test steel.

First, the determination of the permeability of the under-test steel was performed using a primary standard developed in the laboratory. The device is based on the single sheet standard set-up [25]. It consists of two coupled coils (primary and secondary) surrounding the under-test steel, and a pair of symmetrical soft magnetic yokes attached on either side of the under-test steel, as shown in Figure 6a. The outer coil with 100 turns, 100 mm long, made of 1mm enameled Cu wire, was used for excitation purposes, while the inner one with 2000 turns, 70 mm long, made of 0.1 mm enameled Cu wire, was used for voltage output detection and monitoring. The pair of soft magnetic yokes (7 ppm carbon soft iron) was employed to provide a closed magnetic loop according to the single sheet tester standard procedure. Permeability was determined by monitoring the peak-to-peak voltage output of the secondary coil of Figure 6a.

After the determination of the permeability of the under-test steel by the single sheet tester, a transfer standard instrument (Figure 6b) was calibrated against the permeability of the under-test steels, i.e., to determine the amount of current required in the excitation coil to obtain a field dependence of permeability identical to that of the primary standard. This instrument is comprised of a Π shaped 7 ppm carbon soft iron electromagnetic yoke, with a 50 mm long excitation coil made of 100 turns of 1 mm enameled Cu wire, and with a 50 mm long detection coil made of 1000 turns of 0.1 mm enameled Cu wire.

Both instrumentation set-ups used the same excitation and detection circuitry. A home-made current amplifier was used to generate the excitation sinusoidal or triangular current with frequency from 0.1 Hz up to 1 Hz and current from 0.1 A up to 30 A, with minimum current steps of 0.1 A. In fact, 0.1 Hz frequency was used in order to avoid eddy current effects and monitor the intrinsic permeability of the under-test steel, while the current amplitude was changing from 0.1 A to 15 A with steps of 0.1 A for the permeability loop measurement. The detection voltage circuit was based on a high-frequency bandwidth operation amplifier with a gain of 400, a buffer, and an Agilent digital 1 GHz bandwidth oscilloscope. No lock-in amplifier was used because of the low excitation frequency, resulting in negligible phase error. This way, the dependence of the peak-to-peak voltage output of the detection coils, proportional to the differential permeability of the under-test steel on the transmitted current, as a function of the excitation field H, provided the μ(H) dependence. Instead of calculating the permeability response, the voltage output was compared with the permeability values of a 0.25 NOES with known permeability dependence following the classic transfer standard method.

**Figure 7:**
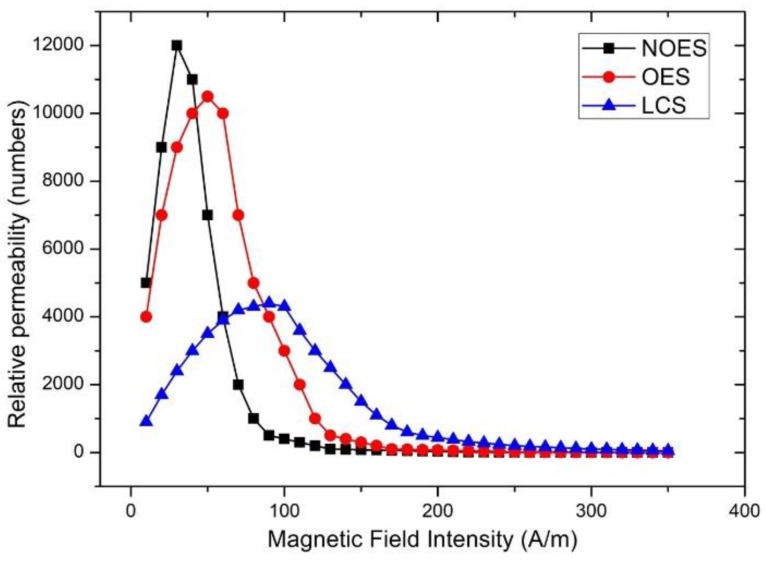
The permeability dependence of three sheets of steel on the excitation field.

The three under-test steel coupons were first tested with the primary single sheet tester. Next, they were tested by the transfer standard. In both cases, the results were identical, with an uncertainty of measurement in the order of 0.01% for the primary tester and less than 0.1% for the transfer standard (Figure 7).

**Figure 8:**
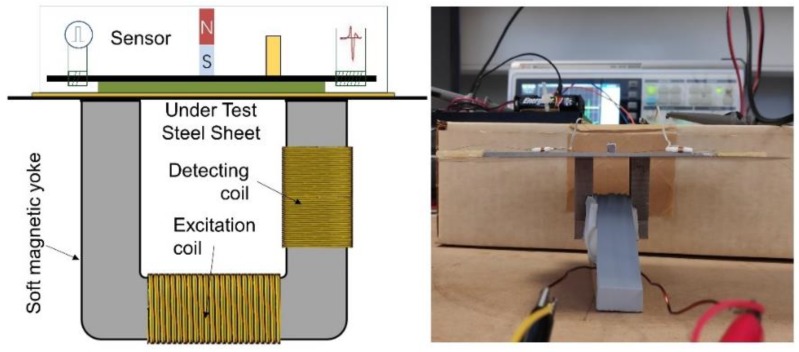
The schematic and photo of the calibration of the sensors with respect to permeability of the under-test steel, determined by the current transmitted to the excitation coil and monitored by the secondary coil of the Π shaped 7 ppm carbon soft iron electromagnetic yoke.

**Figure 9:**
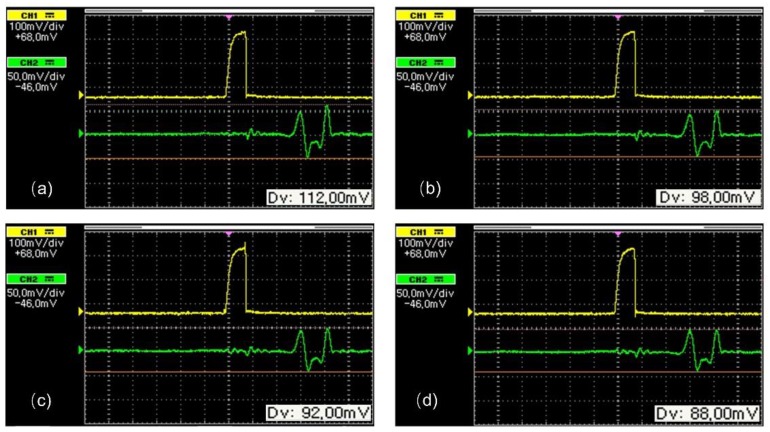
The response of the sensor of Figure 4a with respect to the permeability of the under-test steel, for various voltage inputs to the Π shaped 7 ppm carbon soft iron electromagnetic yoke: (**a**) 1 V; (**b**) 5 V; (**c**) 8 V; (**d**) 10 V.

**Figure 10:**
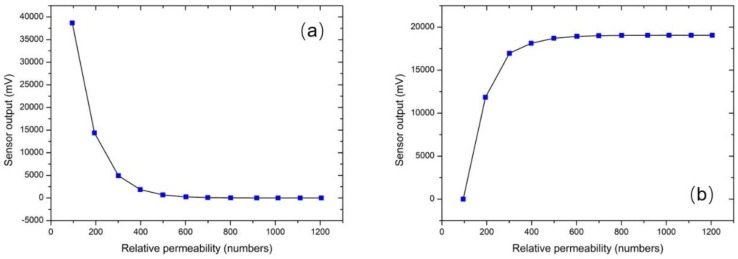
The response of the force sensor based on the MDL technique, for all three samples: (**a**) the response of the sensor illustrated in Figure 4a; (**b**) the response of the sensor illustrated in Figure 4b.

After the calibration of the transfer instrument, the calibration of the sensors took place, by means of determining the dependence of the peak amplitude of their voltage output, Vo, on the permeability of the under-test steel sheet, controlled by the excitation current in the transfer standard. A schematic of this arrangement is illustrated in Figure 8. The transfer instrument, namely the Π shaped iron electromagnetic yoke, was set at the one side of the under-test steel, while the force sensor on the other one. The 0.1 Hz excitation current in the excitation coil of the yoke was varying from 0.1 A–15 A, in steps of 0.1 A, resulting in different permeability values, determined by the detection coil and compared against the reference values obtained during the calibration phase. The permeability values, as measured by the detection coil of the transfer instrument, were used as input to the force sensor, while the peak output voltage of the search coil of the force sensor, Vo, was its output. Figure 9 shows the response of the sensor with respect to permeability of the under-test steel for various excitation voltage levels. The effect of the magnetic field emanating from the permanent magnet is considered as offset. Figure 10 illustrates the response of the force sensor depicted in Figure 4a on the permeability of the three under-test sheets. The response of the force sensor with respect to the monitored permeability from the three steel coupons was identical with an uncertainty of 1%. Similarly, the response of the force sensor depicted in Figure 4b is illustrated in Figure 10b for all three different types of under-test steels. Again, the response of all steels was identical, with an uncertainty of 0.5%. However, the correlation of the permeability of each steel is not the same for the stress determination, but dependent on their specific magnetic stress calibration curve (MASC) [9]. The hysteresis of the force sensor was negligible as expected, due to the negligible hysteresis of the MSE and the force digitizer. The lift-off effect was manually arranged.

Following the calibration of the force sensor dependence on each sample’s permeability, the stress monitoring procedure using the MASC technique [9] was realized in stress coupons. The values of permeability are correlated to residual stresses in given areas of the surface of non-oriented electric steel (NOES) and AISI 1008 steel welded coupons, using the MASC curves of each material. The stress distribution in these coupons was obtained by autogenous welding using either Plasma or TIG or induction heating welding process, in order not to add new material in them. The amplitudes of stresses, concerning the fusion zone (FZ), the heat affected zone (HAZ), and the base material (BM), are known from previous measurements [9]. XRD-BB tests have been selectively performed at given points of the coupons across the weld, validating the results obtained via permeability measurements. 

**Figure 11:**
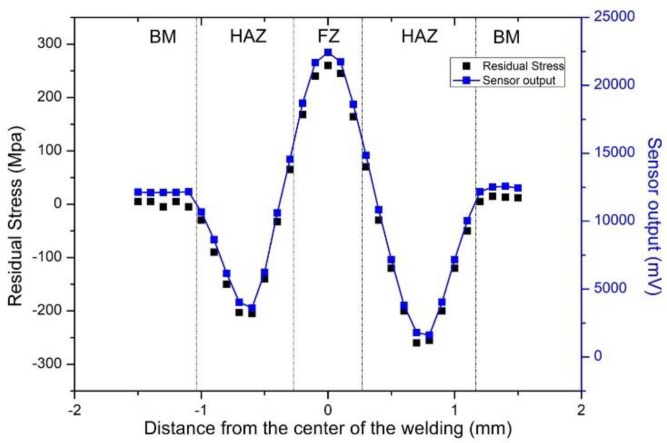
A typical dependence of a welded under-test steel: stress and permeability monitoring across the weld of an AISI 1008 steel. The agreement between stress and permeability distribution is evident.

In this work, the methodology followed for these measurements was as follows: permeability measurements were made by the sensor of Figure 4a at the points where the XRD-BB stress measurements had been performed. The sensor was moved on top of the steel coupon, knowing the position of the middle of the sensing element with an uncertainty of ±0.1 mm. A typical dependence of the response of the force sensor of Figure 4a and the stress components on the same points across the weld is indicatively illustrated in Figure 11 for the AISI 1008 coupon. The agreement between magnetic permeability and stress is evident. Deviation from mean values yielded an uncertainty in the order of 2% with a confidence level of 95.6%. These results permit the use of the force sensor in industrial applications. 

The effect of the ambient magnetic field was studied next: soft iron electromagnet bars were used to apply a magnetic field up to 100 μT in the vicinity of the sensing element in all possible directions. The changes in the sensor output were not detected by the sensor and its 12-bit A/D converter. This, as mentioned already, can be attributed to the strength of the magnetic field from the permanent magnet and the small distance between the permanent magnet and the under-test steel.

The repeatability of the sensor response is mainly due to the negligible hysteresis of the sensors: the measurement at each point does not change when the sensor is moved back and forth. Apart from that, the relatively thick aluminum plate does not permit inclinations of the sensor in order to change the magnetic flux penetration into the under-test steel, therefore maintaining the exerted force between the two magnetic materials. 

The time response of the sensor is practically dependent on the period of the pulsed excitation current, transmitted to the excitation coil plus the time needed for stabilization of the flux inside the under-test steel and the time needed for signal conditioning and transmission. Considering that the period of the pulsed current is 1 ms and the fact that the time for flux stabilization and signal conditioning and transmission is much less than another 1 ms, we can conclude that the response time is in the order of 4 ms. Bearing in mind that the sensing element size is in the order of 5 × 5 mm^2^, the sensor can monitor stress components every 4 mm, with each measurement lasting for 4 ms. Therefore, it can be concluded that the speed of stress monitoring can be up to 4/4 mm/ms or 1 m/s, which is considered to be a fast measurement. Enlarging or lowering the cross-section of the permanent magnet changes the speed of stress monitoring: Lowering the cross-section of the permanent magnet down to a minimum of 1 × 1 mm^2^ decreases the maximum speed of stress monitoring down to 0.25 m/s while improving the spatial resolution to 1 mm^2^, while increasing the cross-section of the permanent magnet (and correspondingly the width of the amorphous magnetostrictive sensing ribbon to an industrially available maximum of 25 mm) increases the maximum speed of monitoring to 6 m/s at the expense of the spatial resolution, which is reduced down to 25 × 25 or 625 mm^2^.

## 4. Discussion and Future Work

The off-vertical orientation of the permanent magnet at the time of gluing or fixing it on top of the MDL, with respect to the under-test steel could cause uncertainties in the measurement of the residual stresses. However, provided that any off-vertical orientation of the permanent magnet is fixed at a given angle, the response of the sensor after performing the calibration steps indicated in Section 3 (Experimental) will follow a given dependence of the sensor output on the permeability of the under-test steel. This actually suggests the obligatory calibration of each sensor before use, based on a standard steel coupon of well-known permeability, which ought to accompany the sensor.

**Figure 12:**
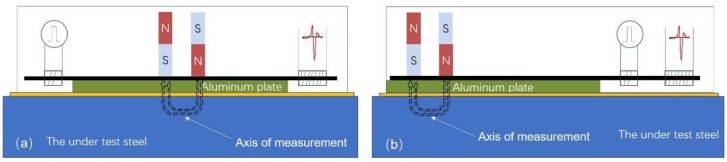
A sensor able to determine the permeability component on the X and Y axes: The magnetic flux between the two permanent magnets is oriented in the main direction; thus, the force exerted on the MDL depends on the permeability along this direction; (**a**,**b**) show two different arrangements of the set-up like Figure 4a,b.

Another issue is the possibility of monitoring the localized spatial components of permeability and, therefore, the localized stress components. The sensor illustrated in this paper is able to monitor the average localized permeability of the under-test steel delimited by the yoke’s poles. To provide the permeability components and, therefore, the stress components of the under-test steel, the sensing elements illustrated in Figure 12 are needed. According to this arrangement, two permanent magnets, parallel to each other are set in opposite orientations normal to the surface of the MDL. Their distance should be large enough to permit a horizontal penetration of their magnetic flux on the surface of the under-test steel. A soft magnetic bar on their free surfaces is preferably set in order to permit magnetic flux closure. Although the modeling of this arrangement is somehow complicated, preliminary experimental results in angular measurements on the surface of the under-test steel illustrated anisotropic dependence of the sensor response on the angle of measurement, when the distance of the two permanent magnets is larger than their length. This is possibly attributed to the different permeability components.

A certain component that the sensor needs for its operation is an automated vehicle to perform automated measurements. The vehicle comprises four wheels, permitting traveling on flat or round surfaces to monitor stresses in base materials, welds, and heat-affected zones. The vehicle can be driven by a power cable and can host the necessary ultrasonic thickness sensor and magnetic rotating encoder. It is preferable that the aluminum chassis is still in touch with the surface of the under-test steel. Otherwise, the lift-off effect must be taken into account and considered for uncertainty, hysteresis, and time response. If the whole sensor is elevated by a fraction of mm from the under-test surface, then the amount of force changes drastically, resulting in significant uncertainties of measurement. Apart from that, geometrical uncertainties may be introduced to the measuring system, thus inviting possible issues of uncertainty. However, the time response seems not to be severely affected by such a lift-off effect. Concerning some non-flat, not-conventional cases of stress monitoring, where the automated vehicle should not be horizontal, magnetic wheels or magnetic caterpillar tracks may be employed in order to get an automated process of stress monitoring. In the event that an autonomous stress monitoring system is required to be developed, the autonomous monitoring vehicle should carry onboard electric motors, battery banks and, possibly, energy harvesting systems. Work may be needed in this direction.

Applications of the force sensor may be used in several industrial sectors. The first implementation is flat and quasi-flat surfaces and steel structures. Ship hulls and their welded areas in shipyards and points of maintenance are one of the possible applications of the force sensor. In this case, the magnetic wheels or caterpillar tracks are necessary. 

Application in round surfaces, like pipelines and vessels, is possible with this type of sensor provided that the geometry (in other words, the diameter of the pipeline) permits an internal check. Along these lines, the hybrid type of miniaturization of the sensor is an essential key point when pipelines are considered. Bearing in mind the necessity of a ~100 mm (or at least 70 mm) long amorphous ribbon, the design of the sensor and the packaging of its electronics must be along the direction of its movement, with particular attention to driving mechanisms, like magnetic wheels.

A gel can be set as an interface between the sensor support and the under-test steel in order to avoid introducing residual surface stresses due to the wear between the sensor support and the steel surface, as well as sacrificing the support. An ultrasonic thickness detector can be set on top of the sensor support to monitor the reflections from the bottom of the sensor support and the bottom of the under-test steel. As soon as there is a contact between the gel and the steel, apart from the first reflections from the sensor support, a second reflection from the bottom of the under-test steel will appear, determining a zero lift-off between the sensor support and the under-test steel. The incremental position of the sensor can be controlled via a linear or circular encoder or a displacement sensor. The recording of the output starts after the ultrasonic sensor detects the second peak-reflection from the bottom of the under-test steel, certifying that the sensing element is in touch with the under-test steel. The digitized peak to peak voltage amplitude, together with the information from the rotating encoder are stored and transmitted to the hard disk of a laptop. Both circuits are microprocessor controlled.

The main foreseen application in production lines concerns steel sheet manufacturing, including cold and hot rolling processes. For this particular case, a force sensor array is required to monitor in parallel a variable width and variable speed production line. In such production lines, after deciding the desirable spatial resolution with respect to the production speed, two or three arrays of force sensors are feasible for a single line stress monitoring process in order to have the cross-checked possibility. The cost of this array sensor can be minimized by using an in-series electric circuit for current excitation and an ASIC (application-specific integrated circuit) chip, including signal conditioning and microprocessor controller, for lower cost of electronics. Apart from that, the mechanics of the sensor, including ribbons, aluminum chassis, and packaging, can be minimized in terms of production and manufacturing costs, implementing hybrid technologies of manufacturing, including additive manufacturing processes. For this application, a monitoring vehicle and the autonomous process are not required: the sensor remains in position and the manufactured steel moves, while the electric energy required for the stress monitoring control is practically the transmitted electric current for generating the microstrains in each individual sensor. Suppose that each individual sensor requires 10 A pulsed current with a duty cycle of 2:1000 (or 20 mA on average) at an impedance of 1 Ohm maximum, the total required energy is 20 mVA per sensor. Thus, considering, for example, a minimum of 625 mm^2^ spatial resolution (corresponding to a maximum width of the amorphous magnetostrictive ribbon of 25 mm, a rectangular cross-section of the permanent magnet 25 × 25 mm and a minimum corresponding spatial resolution of 25 × 25 mm^2^) for a production line of 600 mm, corresponding to a typical steel sheet manufacturing line, one would need 25 sensors to form a linear array sensor, with another four linear arrays to fulfill the stress monitoring needs. Therefore, 100 sensors are totally needed corresponding to 2 VA required power for the whole system. On the other hand, if a spatial resolution of 1 mm^2^ is needed (the other extreme case of the optimum spatial resolution of 1 × 1 mm^2^) in the same production line, then at least 600 sensing elements are needed for one line check. In order to improve the speed of stress monitoring, a total of 3000 to 5000 sensors may be needed, thus requiring about 100 VA in total, which is still pretty affordable for a production line quality control system.

Another important application may be in transportation, with an emphasis on railways and ships. In both systems, these sensors may significantly aid in the safety and security of both transportation means by monitoring the stress distribution in railway rails and in the ship’s hull, respectively. Both monitoring needs are currently required by railway companies and maritime societies, respectively. In contrast to the production lines, the operation of these sensors in both applications should be realized in an autonomous manner, without using grid facilities. In railways, this is feasible by means of taking advantage of the vertical displacement of the railway lines when a train passes by, which can be in the order of cm, permitting magnetic harvesting due to the vertical vibration movement of a permanent magnet, set inside a coil vertical to the railway rail in the vicinity of the rail interconnection. The energy harvesting at each intersection of rails, where there is a certain need for stress monitoring, can be in the order of several mW, thus permitting autonomous stress monitoring. Similarly, in deep-sea vessels, this type of monitoring is required for approximately 180 points of care of the hull. For such a ship, a similar harvesting technology can be used, taking advantage of the vertical, lateral, and forth-back motion of the ship. In any case, in both transportation systems, a sensor network can be developed based on this sensor, able to provide big data management and inform all involved stakeholders of the health monitoring of either railways or ships.

Other applications may also be determined, requiring the improvement of the sensor itself as well as the systems operating around it. An example is the case of seamless tube steel heat exchangers, where stress monitoring is required in every infinitesimal part of the heat exchanger in order to avoid undesirable cracks and failures, requiring a stop of the production line for a longer time. Another application can be the deterioration of austenite steel structures, whereby the non-magnetic austenite, before its failure, suffers phase transformation to martensite, which is magnetic. In this particular case, any magnetic response from the austenite, detectable by the force sensor, is translated to the forthcoming failure, requiring urgent maintenance.

## 5. Conclusions

A sensor based on the magnetostrictive delay line technique, able to monitor the force exerted between a permanent magnet and a steel under test is presented in this paper. The force is dependent on the permeability of the under-test steel and therefore on the localized residual stresses, following the MASC technique. The uncertainty achieved is under 2%, while the spatial resolution may vary from 1 mm up to 25 mm, depending on the monitoring speed desired. The sensor can be used to monitor stress in critical steel structures, steel production, and manufacturing. Future work will be dedicated to its optimization through the parametric study and model of the sensor’s response.
